# Supplementary material for: Convergent evolution of hexenal isomerases in Lepidoptera and plants
Source: Nat Ecol Evol. 2026 Feb 27;10(4):807–19. doi: 10.1038/s41559-026-02999-2 (PMC13076199; doi:10.1038/s41559-026-02999-2)
Supplement: Supplementary file 1 — Supplementary Figs. 1–9. [file 41559_2026_2999_MOESM1_ESM.pdf]

nature ecology & evolution

Supplementary information https://doi.org/10.1038/s41559-026-02999-2

Convergent evolution of hexenal isomerases in Lepidoptera and plants

In the format provided by the authors and unedited

Supplementary Information

Table of Contents

Supplementary Fig. 1….p.2-3
Supplementary Fig. 2….p.4-5
Supplementary Fig. 3….p.6
Supplementary Fig. 4….p.7
Supplementary Fig. 5….p.8
Supplementary Fig. 6….p.9
Supplementary Fig. 7….p.10
Supplementary Fig. 8….p.11
Supplementary Fig. 9….p.12
References….p.13

Supplementary Fig. 1. Detailed tree of lepidopteran GMC oxidoreductases with accession numbers and taxon.

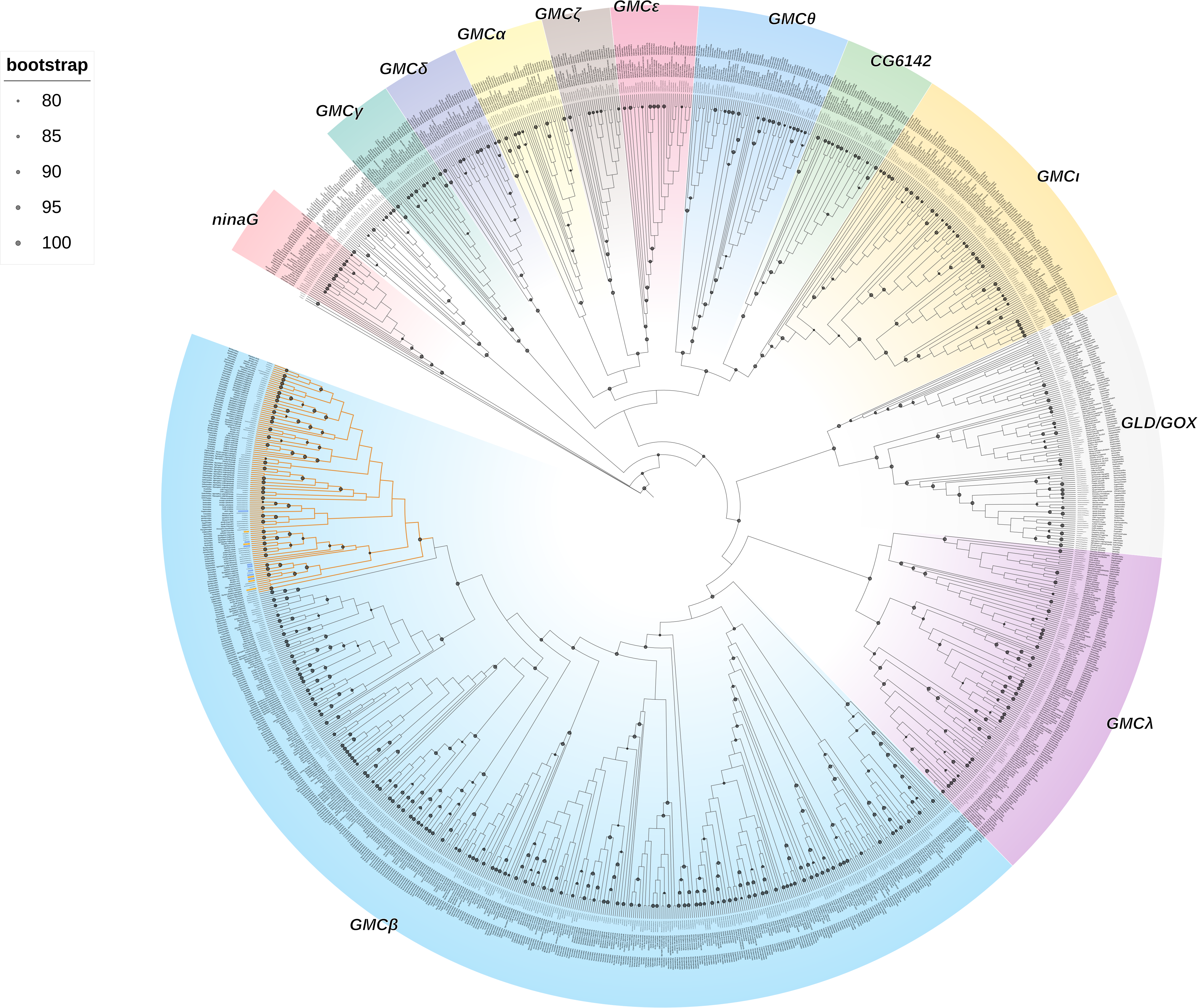

bootstrap
80
85
90
95
100
GMCγ
GMCδ
GMCα
GMCζ
GMCε
GMCθ
CG6142
GMCι
GLD/GOX
GMCλ
GMCβ
ninaG

Supplementary Fig. 2. Focused tree of the putative Hi clade within the GMCβ subfamily.

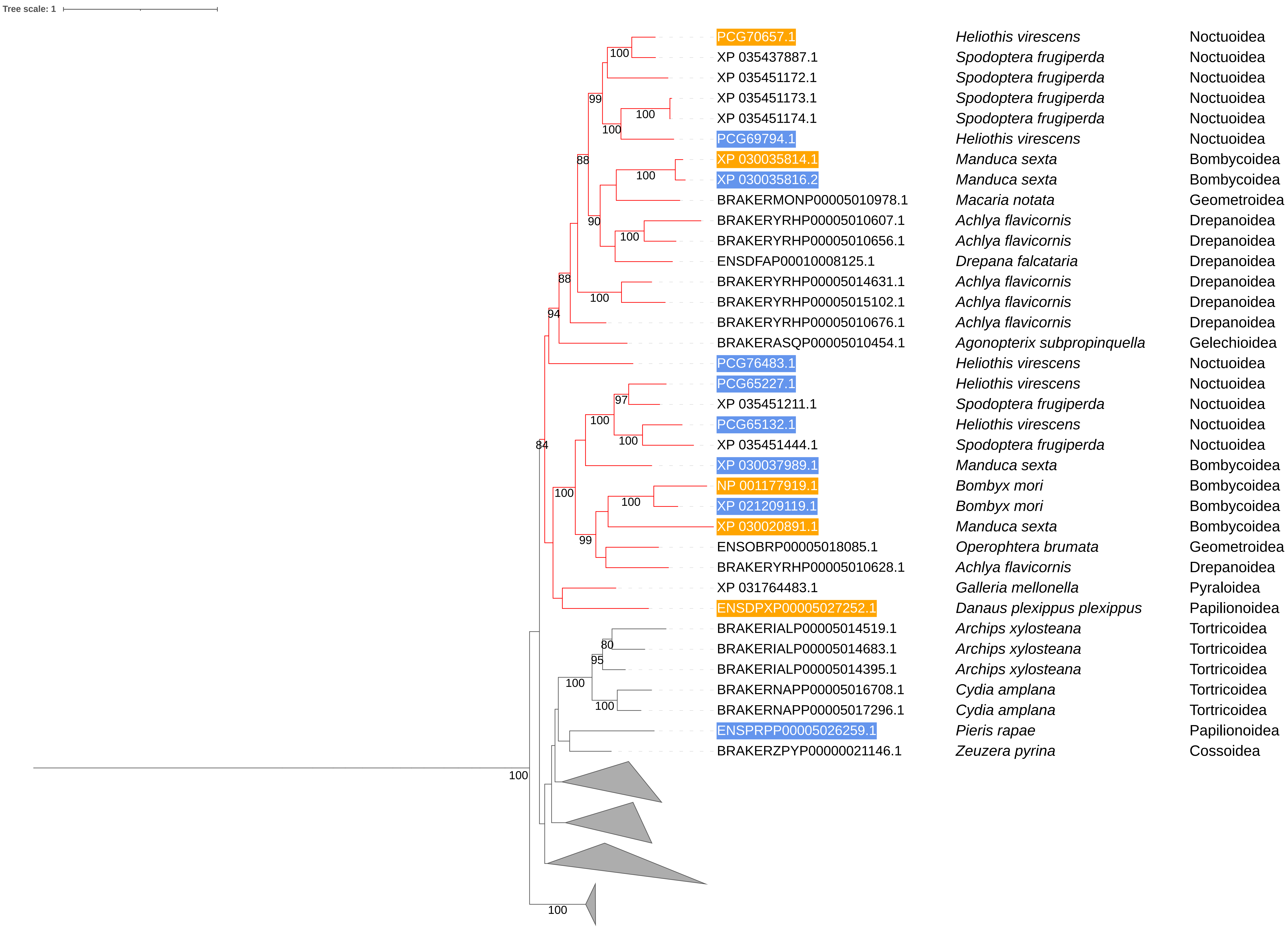

Tree scale: 1
PCG70657.1 Heliothis virescens Noctuoidea
XP 035437887.1 Spodoptera frugiperda Noctuoidea
XP 035451172.1 Spodoptera frugiperda Noctuoidea
XP 035451173.1 Spodoptera frugiperda Noctuoidea
XP 035451174.1 Spodoptera frugiperda Noctuoidea
PCG69794.1 Heliothis virescens Noctuoidea
XP 030035814.1 Manduca sexta Bombycoidea
XP 030035816.2 Manduca sexta Bombycoidea
BRAKERMONP00005010978.1 Macaria notata Geometroidea
BRAKERYRHP00005010607.1 Achlya flavicornis Drepanoidea
BRAKERYRHP00005010656.1 Achlya flavicornis Drepanoidea
ENSDFAP00010008125.1 Drepana falcataria Drepanoidea
BRAKERYRHP00005014631.1 Achlya flavicornis Drepanoidea
BRAKERYRHP00005015102.1 Achlya flavicornis Drepanoidea
BRAKERYRHP00005010676.1 Achlya flavicornis Drepanoidea
BRAKERASQP00005010454.1 Agonopterix subpropinquella Gelechioidea
PCG76483.1 Heliothis virescens Noctuoidea
PCG65227.1 Heliothis virescens Noctuoidea
XP 035451211.1 Spodoptera frugiperda Noctuoidea
PCG65132.1 Heliothis virescens Noctuoidea
XP 035451444.1 Spodoptera frugiperda Noctuoidea
XP 030037989.1 Manduca sexta Bombycoidea
NP 001177919.1 Bombyx mori Bombycoidea
XP 021209119.1 Bombyx mori Bombycoidea
XP 030020891.1 Manduca sexta Bombycoidea
ENSOBRP00005018085.1 Operophtera brumata Geometroidea
BRAKERYRHP00005010628.1 Achlya flavicornis Drepanoidea
XP 031764483.1 Galleria mellonella Pyraloidea
ENSDPXP00005027252.1 Danaus plexippus plexippus Papilionoidea
BRAKERIALP00005014519.1 Archips xylosteana Tortricoidea
BRAKERIALP00005014683.1 Archips xylosteana Tortricoidea
BRAKERIALP00005014395.1 Archips xylosteana Tortricoidea
BRAKERNAPP00005016708.1 Cydia amplana Tortricoidea
BRAKERNAPP00005017296.1 Cydia amplana Tortricoidea
ENSPRPP00005026259.1 Pieris rapae Papilionoidea
BRAKERZPYP00000021146.1 Zeuzera pyrina Cossoidea

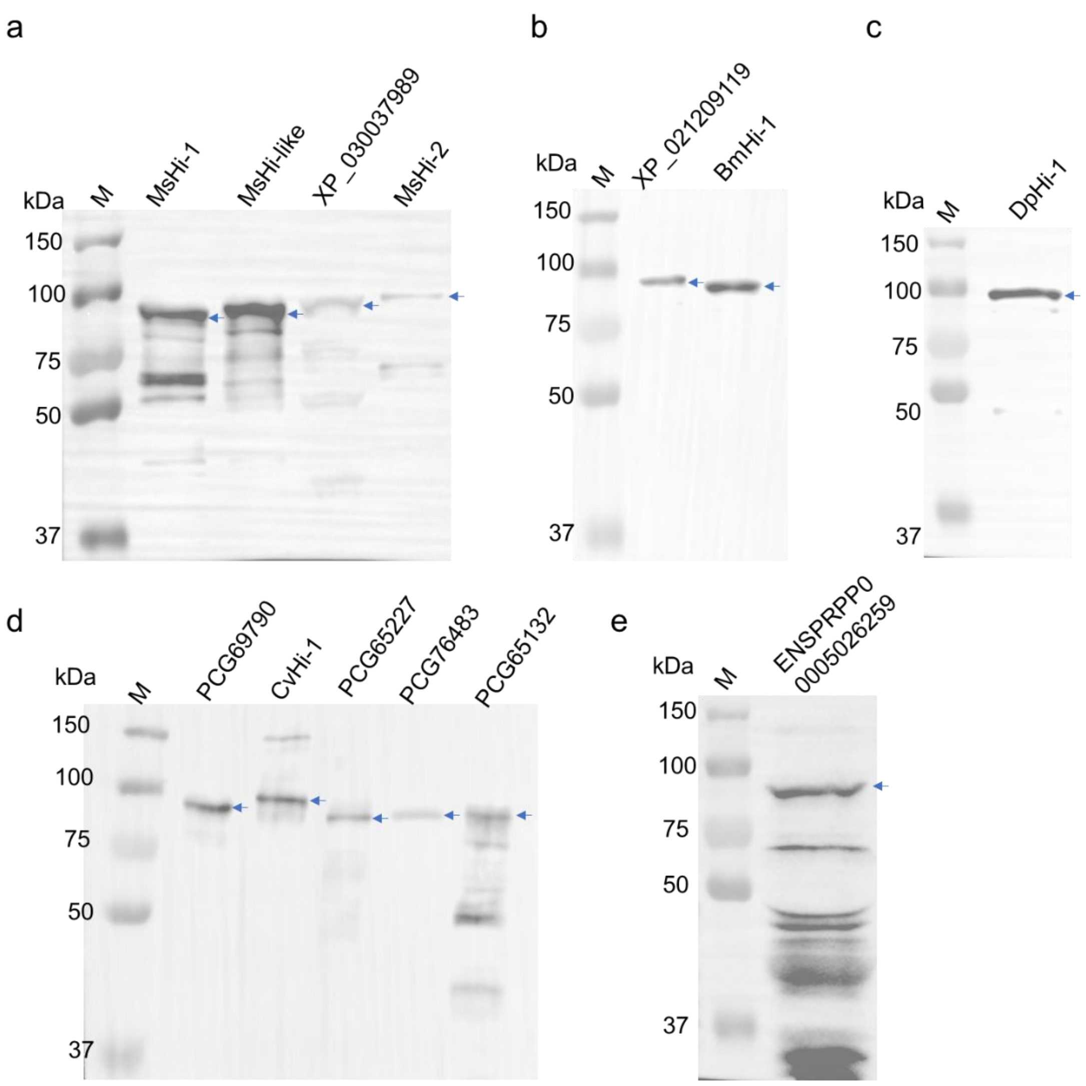

Supplementary Fig. 3. Western blot of recombinant putative Hi expressed in *E. coli* BL21. Genes encoding putative Hi were derived from (a) *Manduca sexta*, (b) *Bombyx mori*, (c) *Danaus plexippus* (d) *Chloridea virescens* and (e) *Pieris rapae*. Blue arrows indicate the position of recombinant Hi. M denotes protein marker.

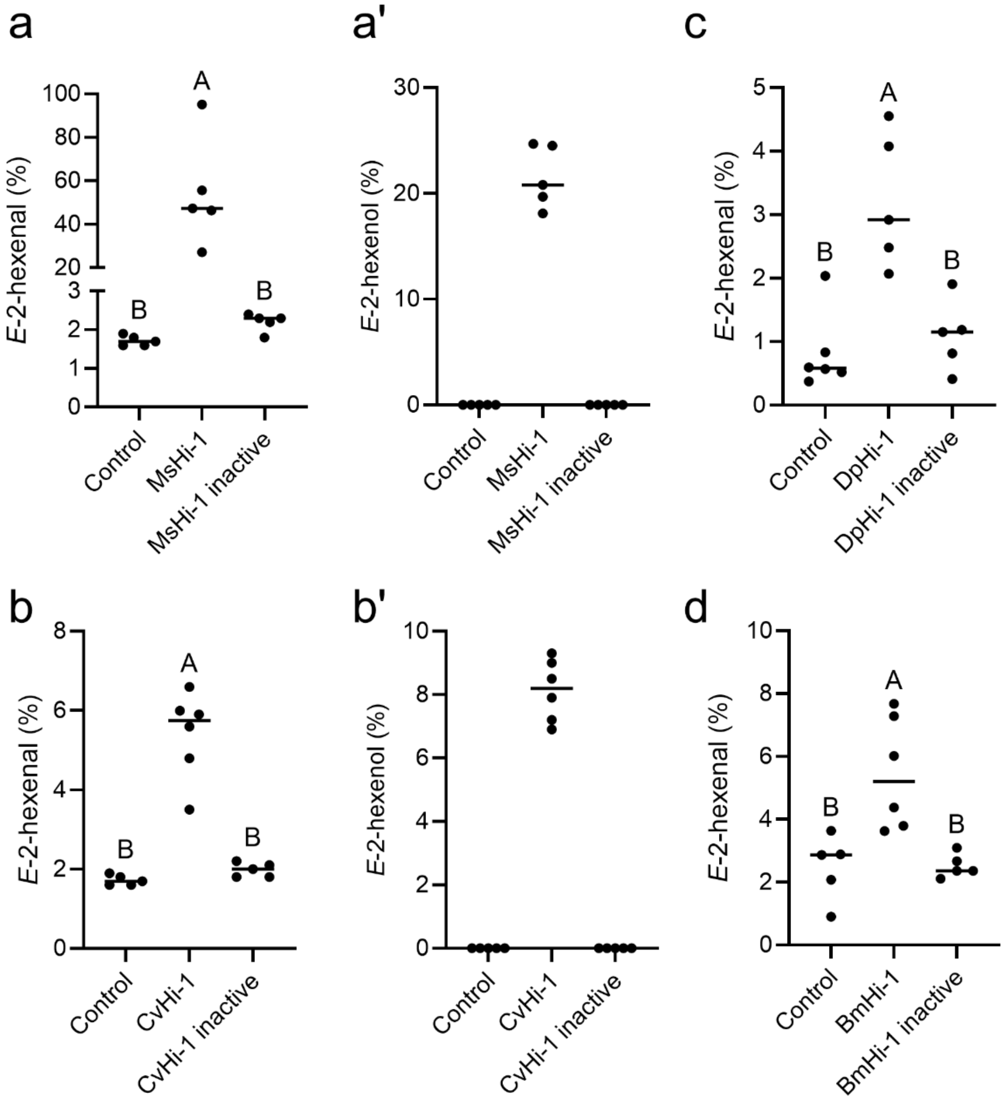

Supplementary Fig. 4. Corresponding dot plot of Fig 3. Lognormal ordinary one-way ANOVA test was performed to assess significant differences between treatments, **a**, $\eta^2 = 0.98$, $F_{2,\,12} = 240$, $p < 0.001$; **b**, $\eta^2 = 0.93$, $F_{2,\,13} = 87{,}26$, $p < 0.001$; **c**, $\eta^2 = 0.65$, $F_{2,\,13} = 12{,}19$, $p = 0.001$; **d**, $\eta^2 = 0.57$, $F_{2,\,13} = 8{,}477$, $p = 0.004$. Different letters on the top of each bar indicate significant differences ($p < 0.05$) by Tukey post-hoc test. n = 5-6 biologically independent samples. The center line indicates median value. Error bars are presented as mean values ± SD. Host plants: (**a**-**b**; **a'**-**b'**) Tomato; (**c**) Milkweed; (**d**) White mulberry.

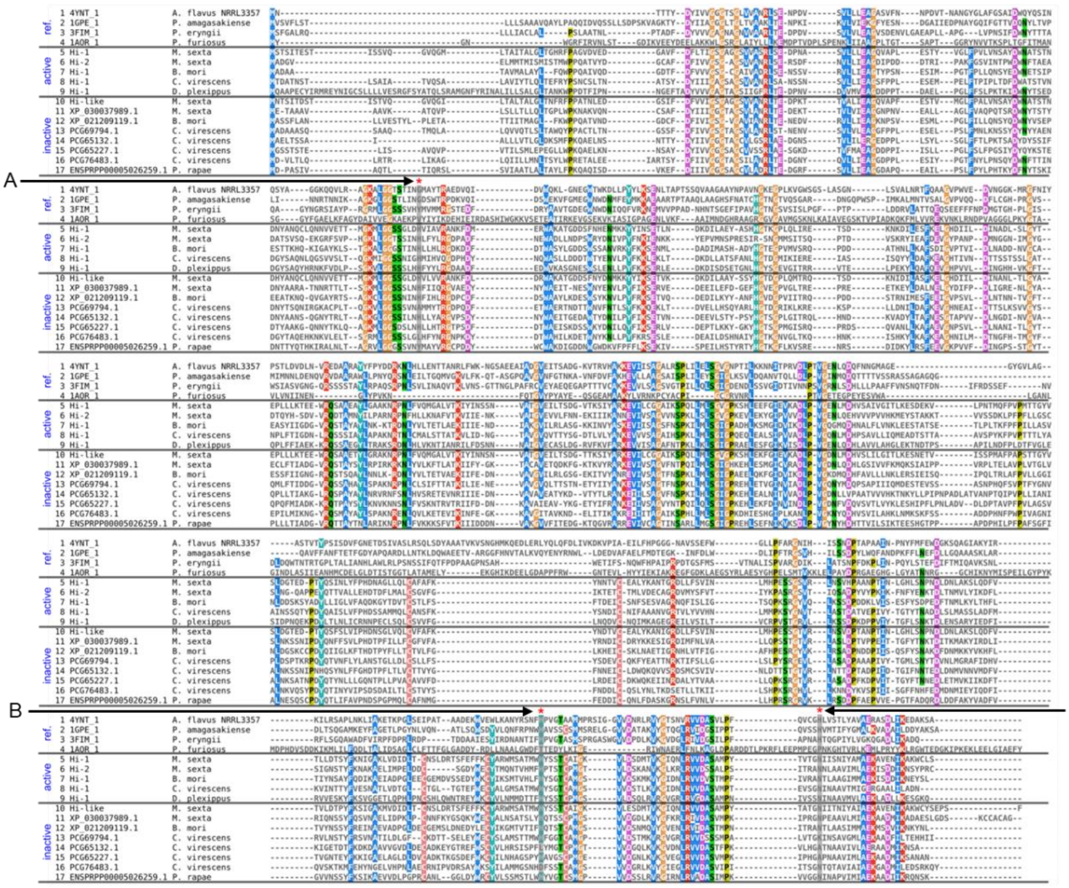

Supplementary Fig. 5. Alignment of Lepidopteran Hi homologs with four reference oxidoreductases. Label A (with a red asterisk) indicates a conserved histidine residue in Lepidopteran Hi proteins. Labels B and C (with red asterisks) denote the conserved catalytic histidine/histidine or histidine/asparagine pairs in GMC oxidoreductase family proteins. Rows 1–3 represent reference GMC oxidoreductases from fungi. Row 4 shows a non-GMC oxidoreductase protein (aldehyde ferredoxin oxidoreductase) from an archaeon. Rows 5–9 correspond to Lepidopteran Hi proteins that exhibit Hi activity, whereas rows 10–17 represent Lepidopteran Hi proteins that do not show Hi activity

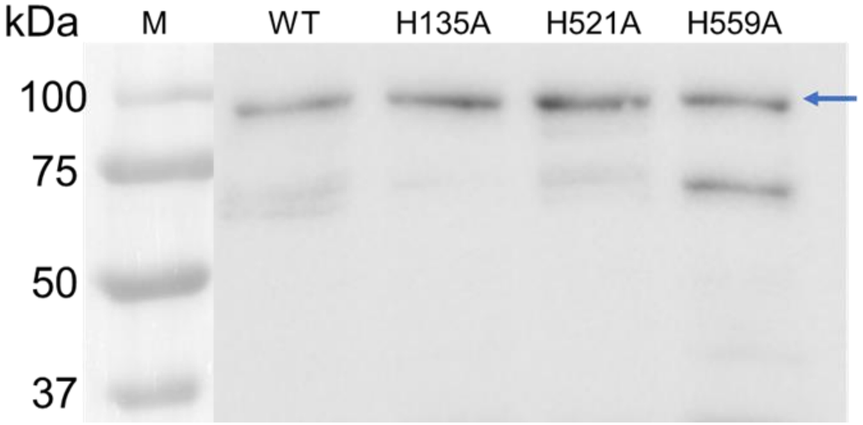

Supplementary Fig. 6. Western blot of recombinant wildtype MsHi-1 (WT) and three alanine-substitution MsHi-1 mutant proteins expressed in *E. coli* BL21. Blue arrow indicates the position of recombinant Hi. M denotes protein marker. The unprocessed image is provided in the Source Data file.

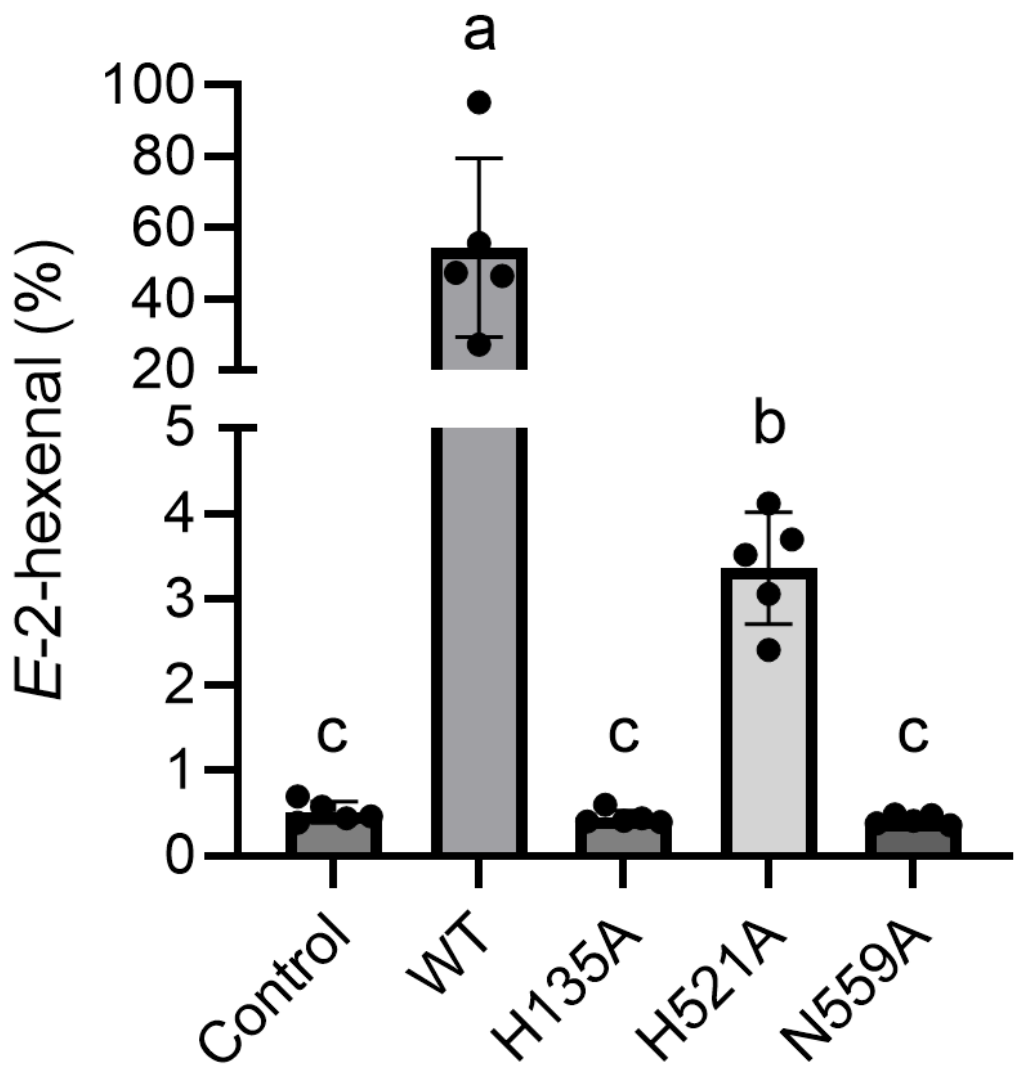

Supplementary Fig. 7. In planta assay for measuring Hi activity in wildtype and mutant MsHi-1 proteins. Leaf discs from tomato were mechanically wounded and treated with 10 µL of Milli-Q water (control), 0.1 µg of wildtype (WT) and three alanine-substitution MsHi-1 mutant proteins. Proportion of *E*-2-hexenal emitted from total aldehydes (*Z*-3-hexenal + *E*-2-hexenal) was calculated. Different letters indicate significant differences (p <0.05) between groups (ANOVA followed by Tukey HSD post-hoc analysis, $\eta^2 = 0.98$, $F_{4,20}$= 307.4, p <0.0001, n = 5). Error bars are presented as mean values ± SD.

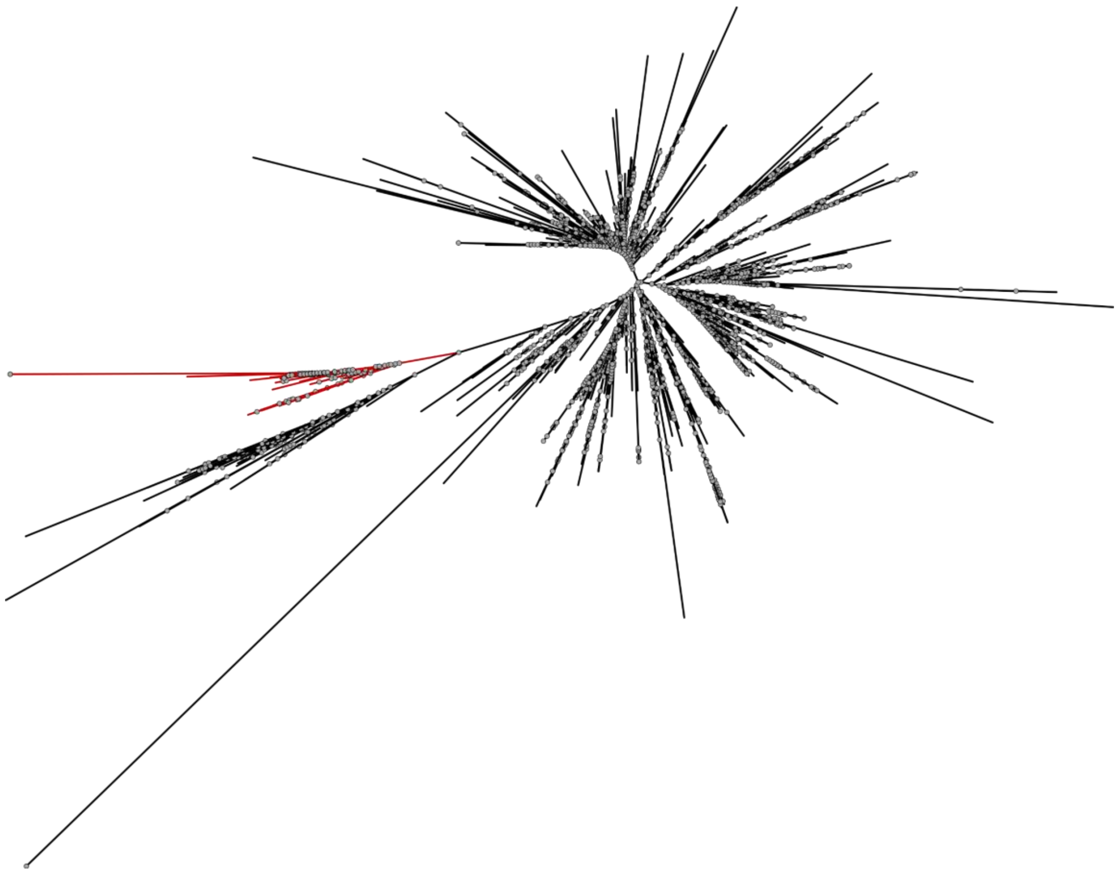

Supplementary Fig. 8. An un-rooted Maximum likelihood (ML) phylogenetic tree of the cupin superfamily. The ML tree was constructed using full-length protein sequences containing the cupin domain (Pfam: PF07883), combined with representative sequences from known cupin superfamily (Germin, Vicilin, Legumin, Globulin, and HI). Branch support values are inferred using IQ-TREE/RAxML-NG. Previously characterized plant HI (Kunishima et al., 2016; Spyropoulou et al., 2017) are clustered in a distinct subclade (highlighted in red).

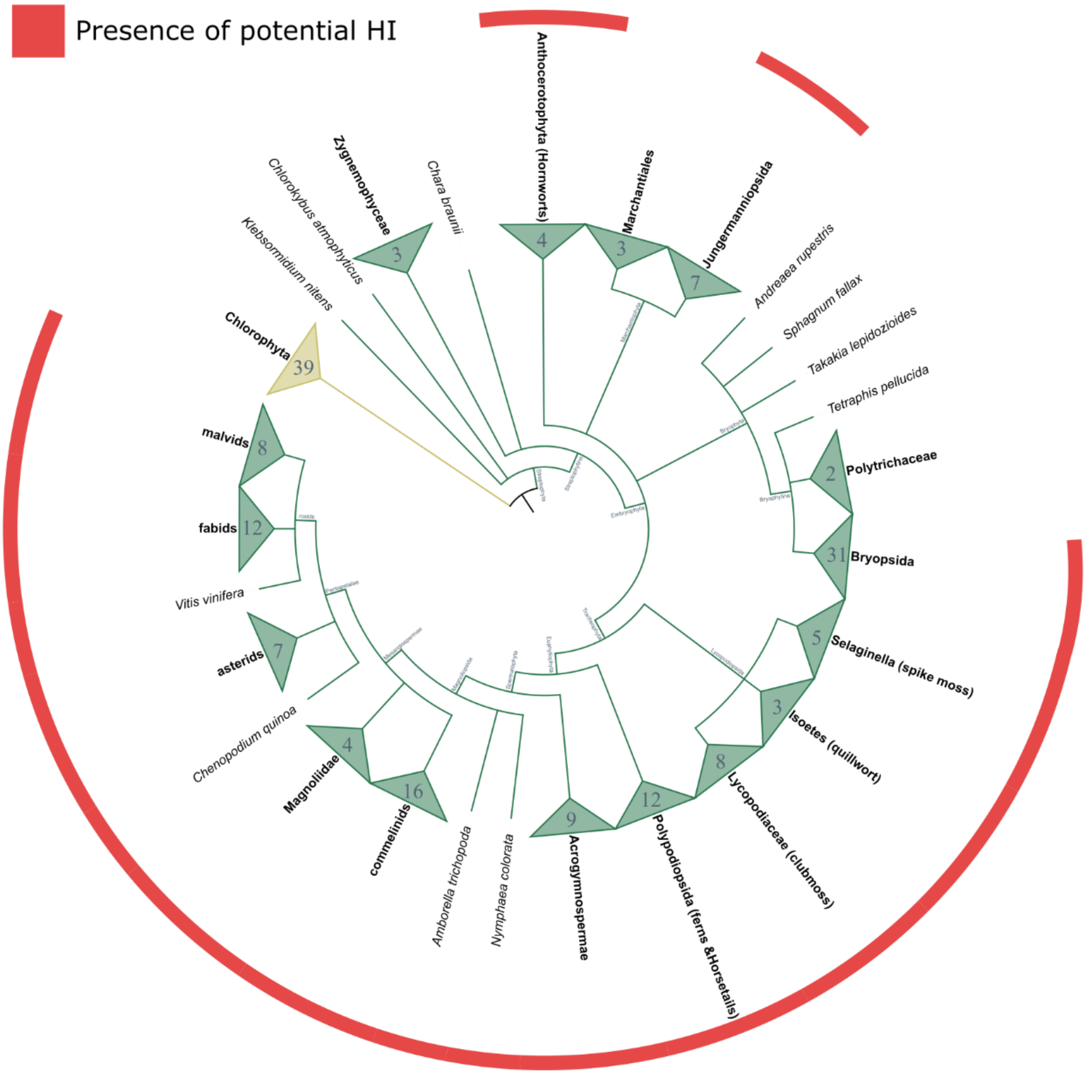

Supplementary Fig. 9. Cladogram showing the diversity of putative HI orthologs across the green lineage (Viridiplantae) representatives. Red lines highlight lineages that possess orthologs belonging to the HI-potential clade. The numbers on branches indicate the number of species represented within each group.

References

Kunishima, M., Yamauchi, Y., Mizutani, M., Kuse, M., Takikawa, H., Sugimoto, Y., 2016. Identification of (Z)-3:(E)-2-Hexenal Isomerases Essential to the Production of the Leaf Aldehyde in Plants. J Biol Chem 291, 14023-14033.

Spyropoulou, E.A., Dekker, H.L., Steemers, L., van Maarseveen, J.H., de Koster, C.G., Haring, M.A., Schuurink, R.C., Allmann, S., 2017. Identification and Characterization of (3Z):(2E)-Hexenal Isomerases from Cucumber. Front Plant Sci 8, 1342.
